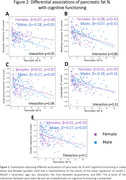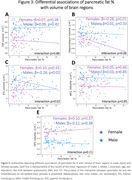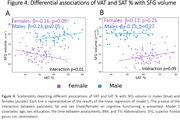# Abdominal Fat Depots are Related to Lower Cognitive Functioning and Brain Volumes in Middle‐aged Males at High Alzheimer’s Risk

**DOI:** 10.1002/alz.086096

**Published:** 2025-01-09

**Authors:** Sapir Golan, Ethel Boccara, Ramit Ravona‐Springer, Yael Inbar, Hila Zelicha, Abigail Livny, Barbara B. Bendlin, Orit H. Lesman‐Segev, Iscka Yore, Anthony Heymann, Mary Sano, Yael Mardor, Joseph Azuri, Michal S Beeri

**Affiliations:** ^1^ Sackler Faculty of Medicine, Tel Aviv University, Tel Aviv Israel; ^2^ The Joseph Sagol Neuroscience Center, Sheba Medical Center, Tel Hashomer Israel; ^3^ Department of Psychology, Bar Ilan University, Ramat Gan Israel; ^4^ Memory clinic and The Joseph Sagol Neuroscience Center, Sheba Medical Center, Israel, Ramat‐Gan Israel; ^5^ Department of Diagnostic Imaging, Sheba Medical Center, Tel Hashomer Israel; ^6^ Ben‐Gurion University of the Negev, Beer‐Sheva Israel; ^7^ The Sagol School of Neuroscience, Tel Aviv University, Tel Aviv Israel; ^8^ Wisconsin’s Alzheimer’s Institute, School of Medicine and Public Health (SMPH), University of Wisconsin‐Madison, Madison, WI USA; ^9^ Icahn School of Medicine at Mount Sinai, New York, NY USA; ^10^ James J. Peters Veterans Affairs Medical Center, Bronx, NY USA; ^11^ Maccabi Health Services, Tel‐Aviv Israel; ^12^ Herbert and Jacqueline Krieger Klein Alzheimer’s Research Center at Rutgers Brain Health Institute, New Brunswick, NJ USA; ^13^ Krieger Klein Alzheimer’s Research Center, New Brunswick, NJ USA

## Abstract

**Background:**

High body mass index (BMI), which poorly represents specific fat depots, is linked to poorer cognition and higher dementia risk, with different associations between sexes. We examined associations of abdominal fat depots with cognition and brain volumes and whether sex modifies this association.

**Method:**

204 healthy middle‐aged Alzheimer’s‐dementia (AD) offspring (mean age = 59.44, 60% females) underwent abdominal magnetic resonance imaging to quantify hepatic, pancreatic, visceral (VAT), and subcutaneous adipose tissue (SAT), assessment of cognition and brain volumes.

**Result:**

In the whole sample higher hepatic fat % was associated with lower total grey matter volume (β = ‐0.17, p<0.01). Primarily in males, higher pancreatic fat % was associated with lower global cognition (Males: β = ‐0.27, p = 0.03; Females: β = 0.01, p = 0.93) executive function (Males: β = ‐0.27, p = 0.03; Females: β = 0.02, p = 0.87), episodic memory (Males: β = ‐0.28, p = 0.03; Females: β = 0.07, p = 0.48) and inferior frontal gyrus volume (Males: β = ‐0.28, p = 0.02; Females: β = 0.10, p = 0.33). VAT and SAT were inversely associated with middle frontal and superior frontal gyrus volumes in males and females.

**Conclusion:**

In middle‐aged males at high AD‐risk, but not in females, higher pancreatic fat, was associated with lower cognition and brain volumes. These findings suggest a potential sex‐specific link between distinct abdominal fat with brain health. By AAIC 2024 300 participants will be scanned, additional brain pathlogies will be assesed (amyloid, tau, etc.) and secreted factors will be evaluated (GLP‐1, Amylin, PAI‐1, leptin, etc). These data may lead to the development of fat depot‐specific interventions, which are expected to prevent or delay brain atrophy and possibly cognitive decline.